# Lessons from case studies of integrating mental health into primary health care in South Africa and Uganda

**DOI:** 10.1186/1752-4458-5-8

**Published:** 2011-04-15

**Authors:** Inge Petersen, Joshua Ssebunnya, Arvin Bhana, Kim Baillie

**Affiliations:** 1 School of Psychology, University of KwaZulu-Natal, South Africa; 2 Institute of Psychology, Makerere University, Uganda; 3 Human and Social Development, Human Sciences Research Council, South Africa and the University of KwaZulu-Natal, South Africa; 4 School of Psychology, University of KwaZulu-Natal, South Africa

## Abstract

**Background:**

While decentralized and integrated primary mental healthcare forms the core of mental health policies in many low- and middle-income countries (LMICs), implementation remains a challenge. The aim of this study was to understand how the use of a common implementation framework could assist in the integration of mental health into primary healthcare in Ugandan and South African district demonstration sites. The foci and form of the services developed differed across the country sites depending on the service gaps and resources available. South Africa focused on reducing the service gap for common mental disorders and Uganda, for severe mental disorders.

**Method:**

A qualitative post-intervention process evaluation using focus group and individual interviews with key stakeholders was undertaken in both sites. The emergent data was analyzed using framework analysis.

**Results:**

Sensitization of district management authorities and the establishment of community collaborative multi-sectoral forums assisted in improving political will to strengthen mental health services in both countries. Task shifting using community health workers emerged as a promising strategy for improving access to services and help seeking behaviour in both countries. However, in Uganda, limited application of task shifting to identification and referral, as well as limited availability of psychotropic medication and specialist mental health personnel, resulted in a referral bottleneck. To varying degrees, community-based self-help groups showed potential for empowering service users and carers to become more self sufficient and less dependent on overstretched healthcare systems. They also showed potential for promoting social inclusion and addressing stigma, discrimination and human rights abuses of people with mental disorders in both country sites.

**Conclusions:**

A common implementation framework incorporating a community collaborative multi-sectoral, task shifting and self-help approach to integrating mental health into primary healthcare holds promise for closing the treatment gap for mental disorders in LMICs at district level. However, a minimum number of mental health specialists are still required to provide supervision of non-specialists as well as specialized referral treatment services.

## Introduction

There is an increasing burden of mental disorders in low to middle income countries (LMICs), which are often co-morbid with physical diseases [1]. In the context of a scarcity of mental health specialists [2], decentralization and integrated primary mental healthcare, embracing a task shifting approach, has been mooted as a mechanism to address the treatment gap for mental disorders in these contexts [3,4]. To this end, an increasing body of evidence attests to the effectiveness of task shifting for specific mental disorders in LMICs. A recent PLoS Medicine series provides a review of evidence-based packages of care [5]. There are also emerging models for integrated packages of care embracing task shifting at district level [6,7].

It is therefore not surprising that decentralized and integrated primary mental healthcare forms the core of many policies in LMICs in Africa [8]. Indeed, at the inception of the Mental Health and Poverty Project (MHaPP), a four-country study focused on mental health policy development and implementation to break the cycle of poverty and mental ill-health, both South Africa and Uganda had draft mental health policies in place to promote integrated primary mental healthcare to varying degrees [9,10]. However, in both country contexts, this did not routinely translate into implementation [9,11]. This mirrors evidence from a recent review of studies on community mental healthcare in the African region which indicates that even where policies to support decentralized mental healthcare exist, implementation remains a challenge [8]. Possible barriers to effective implementation suggested by Flisher et al. [12] include: (i) that policy objectives may be unrealistic given available resources; (ii) there may be a lack of appropriate delivery systems to support the policy; and (iii) there may be insufficient support for the policy at the implementation level.

The MHaPP initially conducted situational analyses of mental health services in Uganda and South Africa nationally as well as within typical case study districts/sub-districts [10,11,13,14]. Based on these findings, the MHaPP then undertook to integrate mental healthcare using a task shifting approach in these case study districts/sub-districts sites as demonstration projects. In the context of Uganda being a low-income country with fewer specialist resources than South Africa, a middle-income country [see Table 1 for a comparison], the foci of the case study demonstration projects differed across the country sites. The focus in the Ugandan demonstration site was on task shifting for severe mental disorders (SMDs). These refer mainly to psychotic disorders, which are chronic and recurrent and result in a high disability burden for sufferers and their families. This focus emerged from the paucity of adequate treatment and care at the primary healthcare (PHC) level for these disorders, with most psychiatric patients seeking treatment directly from secondary or tertiary levels of care [10,14]. In contrast, decentralization efforts by the Department of Health in post-apartheid South Africa have focused largely on SMDs [11,13]. There remains, however, a large treatment gap for common mental disorders (CMDs) [15], referring mainly to anxiety and depressive disorders which are less easily identifiable and often present as physical complaints in PHC settings in LMICs. In the context of depression having the highest 12-month prevalence for any individual disorders in South Africa [16], integration and task shifting efforts in the South African site focused on depression. These different foci reinforce the need for a contextually driven approach to integration of mental health into primary healthcare as suggested by the World Health Organization (WHO) 2001 World Health Report [17]. The WHO [17], suggests that the reach of care provided should be dependent on resources available. South Africa, being a medium resourced country, has more resources than Uganda (see Table 1) to warrant an expansion of mental health services to include treatment for CMDs.

**Table 1:** Comparison of gross domestic product and mental health resources per population ratio for Uganda and South Africa

Country characteristics	Uganda	South Africa
Population [35]	33 398 692	49 109 107

Gross domestic product (purchasing power parity) in 2010 [35]	US $ 41.7 billion	US $ 527.5 billion

Psychiatric nurses/100 000 [2]	2.0	7.5

Psychiatrists/100 000 [2]	1.6	1.2

Psychologists in mental health/100 000 [2]	2.0	4.0

Psychiatric beds/population ratio	3.65 psychiatric beds/100 000 [10]	27.9 psychiatric beds/100 000 [11]

A common implementation framework that embraced a multi-sectoral community collaborative, task shifting and self-help approach was used across both country sites in the implementation phase of the MHaPP. This framework was flexible enough to accommodate the various resource constraints and intervention priorities of the different country scenarios. Table 2 outlines the implementation framework and provides a summary of the activities undertaken in the two country sites during the two-year intervention phase of the project (2008-2009). As reflected in Table 2, the implementation framework comprised: (i) reorientation of district management towards integrated primary mental healthcare; (ii) establishment of community collaborative multi-sectoral forums; (iii) task shifting which entailed establishing an expert consultancy liaison mental health team and training of general PHC staff and community health workers (CHWs) or equivalents in identification, management and referral of mental disorders; and (iv) promotion of self-help groups at the community level. In the South African site, an additional component of task shifting was the training of 2 dedicated community mental health workers (CMHWs) to provide a specific psychological treatment, namely, an adapted version of group interpersonal therapy (IPT) under the supervision of a mental health counsellor.

**Table 2:** Implementation framework and activities across the two country sites

Interventions	South Africa	Uganda
Reorientation of district management.	Regular sensitization and feedback meetings were held with district management throughout the duration of the project.	Regular sensitization workshops and feedback meetings with district management throughout the project.

Establish a community collaborative multi-sectoral forum.	Established - met 3-4 times a year.	Established - met twice a year.

Establish an expert mental health consultancy liaison team to provide support and supervision of primary healthcare personnel,	1. Two additional Psychiatric Nurses dedicated to providing support to nurses at the PHC clinics were deployed by the sub-district health authority. 2. A permanent position for a Psychologist was created by the sub-district health authority. 3. Consolidation of services of a consultant Psychiatrist was obtained by the sub-district health authority. 4. A Mental Health Counsellor was employed by the project for the duration of the project to provide training, support and supervision to CHWs and a referral service at PHC clinic level	1. Regular supervisory visits to the health centres by regional support supervision team was facilitated. This team- comprised a Psychiatrist and 2 Psychiatric Clinical Officers. 2. The specialist Mental Health Nurse in the district rotated through the health centres on a regular basis, providing supervision and support to general healthcare workers.

Manualized training of general health workers and CHWs/equivalents in identification, management and referral of persons with mental health problems.	1. Week long training of 12 PHC nurses from PHC clinics in the sub-district in identification, management and referral of mental disorders. 2. CHWs (30) servicing sub-district DSA exposed to 4 day training workshops (2) in identification of CMDs, supportive counselling and problem management skills. 3. Two additional dedicated community mental health workers (CMHWs) trained to run a specific psychological treatment for depression which was an adapted manualized version of group Interpersonal Therapy (IPT).	1. Week long training of general health workers of various cadres (Medical Officer, Clinical Officers, Nurses, Midwives and Nursing Assistants) (150) in identification, management and referral of mental disorders, especially severe mental disorders 2. Sensitization and training workshops in identification, management and/or referral of severe and more common mental disorders for CHWs and community leaders in the 3 sub-districts were held.

Development of community-based self-help groups	At least three self-help groups for people with CMDs formed by CHWs • Provided supportive counselling • Income generating projects	User-carer group comprising approximately 200 families of severe mental disorders formed. • Met once a month • Assisted users to access medicine • Income generating activities initiated - piggery farming, chicken rearing and rice growing • Saving bank for medication initiated

The aim of this study was to understand how the use of the common implementation framework assisted in the development of district/sub-district mental health services in the two country contexts with the view to drawing out shared lessons for integrating mental health into PHC in LMICs.

## Methodology

### Description of intervention sites

The study sites in both countries were chosen on the basis of being rural underserved districts/sub-districts as well as being internationally recognized Demographic Surveillance Areas (DSAs). Demographic and health data are collected on a regular basis in DSAs with the view to monitoring and tracking the health status of household respondents. Consequently, these areas are well described. They also provide research infrastructure for action oriented research aimed at testing and evaluating health interventions [18]. They thus provided ideal settings for the two district demonstration projects.

#### South Africa

The South African case study site was located in the Hlabisa sub-district of the Umkhanyakude district in northern KwaZulu-Natal, on the eastern seaboard of South Africa. The area is typical of most rural areas in South Africa, incorporating township, peri-urban areas as well as more remote rural areas. The sub-district has a total population of 225 000 people, with most of the project activities confined to the DSA within the sub-district. The DSA area had a population of 85 000 resident and non-resident people at the time of the investigation and was serviced by 6 primary healthcare clinics linked to a sub-district hospital [19]. At the time of the study there was initially one psychiatric nurse dedicated to mental health. There was also a community service post for clinical psychologists that was filled on an erratic basis. These posts provide for a mandatory one year community service for clinical psychologists on completion of their training in South Africa.

#### Uganda

The study site in Uganda was Mayuge, a predominantly rural district bordered by Iganga in the North, Jinja in the West, Bugiri in the East and Lake Victoria in the South. It is 4,672.22 km^2 ^of which 77% is water and 23% land. The 2007 population estimate for the district was 389,022 [20]. The area has high levels of poverty, a higher birth rate than the national average and the majority of households depend on agriculture as their major economic activity [21]. The district is served by a non-Government hospital (Buluba Hospital) and has 38 PHC facilities serviced by 212 PHC workers. At the time of the study there was one specialist mental health nurse serving the state facilities with one other employed by Buluba Hospital.

### Data collection and sample

Qualitative process evaluation interviews were held with various stakeholders across the two country sites. Semi-structured interviews and focus group discussions were held with key informants involved in the district level interventions including managers, service providers and service users. To ensure that similar data was collected across the country sites, generic interview schedules were developed for each stakeholder group and adapted by the country partners to ensure country specific contextual appropriateness.

In South Africa, four focus group interviews were held with a voluntary sample of 15 community health workers (CHWs) who had received the training described in the implementation framework (see Table 2). In South Africa, CHWs are community members who receive minimal training to provide health education and home-based care through a home visitation programme. They comprise both volunteers and people who receive a stipend for their time through a government contracted non-governmental organization (NGO). All CHWs who received training through the project fell within the latter group, namely, they were receiving a stipend. Individual interviews were held with: (i) two dedicated community mental health workers (CMHWs), who were community members trained and supervised specifically to facilitate IPT groups for depressed women (they were not part of the general CHW programme and received an equivalent stipend through the project); (ii) nine service users of the IPT groups; (iii) four PHC nurses, who serviced the PHC clinics and have a 3-4 year post-secondary school qualification; (iv) two psychiatric nurses deployed to provide a specialist psychiatric service to the area during the lifespan of the project; (v) the mental health counsellor, a specialist cadre of mental health worker with a four-year B.Psych qualification employed by the project for the duration of the intervention to supervise and support the CHWs and CMHWs; (vi) health managers including the provincial community mental health coordinator, who was a psychiatric nurse, the sub-district director who was a senior nursing sister; and (vii) two community representatives on the community collaborative multi-sectoral forum, which comprised representatives from the health, education and social development sectors as well as community leaders and service users.

In Uganda, focus group discussions were held with: (i) users and carers (3); (ii) selected members of the multi-sectoral forum (1); and (iii) CHWs who had received basic training in mental health (1). In Uganda, CHWs are also community members with minimal training and, in the study site, were all volunteers. Individual interviews were held with the following: (i) health managers including the District Health Officer (DHO), District Health Inspector, District Drug Inspector, District Nursing Officer, and the district mental health focal person who was a Medical Clinical Officer; (ii) the psychiatric nurse employed by the state health service; (iii) two general nurses; (iv) two Medical Clinical Officers; and (v) two carers. Three sets of minutes from multi-sectoral forum meetings were also included in the data set from Uganda.

### Data analysis

Interviews were recorded and transcribed verbatim, with those conducted in local languages translated into English and back-translation checks applied by an independent bilingual English-local language speaker to ensure correctness of the translations [22].

The transcribed interviews as well as minutes were analyzed thematically with the assistance of NVIVO8 using the framework approach [23,24]. This approach incorporates five stages of familiarization, development of a thematic framework, coding or indexing, identification of themes or charting and interpretation [23,24]. A coding framework using the implementation framework outlined in Table 2 was developed and country specific data analysed separately. In addition, open coding was used in the identification of additional themes.

### Ethics

Ethical approval was obtained from the University of KwaZulu-Natal Research Ethics Committee in South Africa, the Ugandan Ministry of Health and Makerere University Faculty of Medicine Research Ethics Committee in Uganda. Informed consent was obtained from each participant prior to the interviews which included information ensuring the anonymity of data, the usefulness of their participation for informing the development of district mental health services, what the interviews would entail as well as confirming the voluntary nature of their participation in the interviews.

## Results

### Reorientation of district management

One of the key activities of the implementation framework (see Table 2) was the reorientation of district management towards the importance of integrating mental healthcare into primary healthcare at district level in order to ensure support at district managerial level.

#### South Africa

In South Africa, heightened awareness and support for strengthening mental health services within the sub-district health sector over the two-year intervention phase of the project was reported:

*But listening to some of the participants particularly from the health sector, one would get that sense that really conscientization and awareness has been heightened as a result of this participation. And one would feel that when these issues were discussed people were passionate about them... They know that they are under-staffed (but) looking at their enthusiasm, one would realize that it did bring about some change in the way people look into the whole issue of mental health within our sub-district (Community leader)*.

In the face of severe budgetary constraints which had resulted in freezing of vacant posts within the Department of Health in the KwaZulu-Natal province of South Africa at the time of the MHaPP district intervention, this heightened awareness nevertheless translated into an actual improvement in the number of human resources dedicated to mental health. This occurred through the deployment of existing psychiatric nursing staff to mental healthcare by the sub-district health manager.

*We have allocated Sister S (an additional psychiatric nurse) to run with mental health... Sister K is also assisting and then of course the psychologist (newly appointed) is helping so there is more representation in general for mental health. Then of course we've also got Sister N who is helping out in the clinics with the mental health side of things (sub-district health manager)*.

#### Uganda

Heightened awareness of the need to increase access to mental health services amongst district managers over the two-year intervention phase of the project was reflected in the following intentions on the part of the District Inspector: (i) to have dedicated days for psychiatric patients for which he could enlist the services of mental health specialists at a regional level; (ii) to develop district mental health plans for some areas; and (iii) a commitment by the District Nursing Officer to recruit at least two mental health nurses during the next recruitment exercise, which at the time of the intervention was expected within 1-2 years.

There was also heightened awareness on the part of health managers of the need to order a sufficient and constant supply of psychotropic medication, which was reported to be more available in the district than it had been prior to the intervention. The supply of medication was, however, erratic, with the district health officer communicating a sense of despair about the situation as reflected in the following quotation.

*For us, what we shall be doing is to order. If (the authorities) can't deliver, we just sit and wait. Because ... sometimes (it) takes long to deliver. For example they were supposed to bring medicines to this region the whole of last week, starting Monday; but they have not yet. Currently we have no drugs at all, in all units. Not only mental health drugs, all drugs (District Health Officer)*.

As reflected in the following excerpt from an interview with the mental health nurse, this resource constraint was strongly de-motivating.

*Because we the health workers are there, we are ready to attend to the patients. But the issue is drugs. You can't serve the community minus drugs... once the drugs are not there, nothing helps...I am one of those who are de-motivated. Because I look at patients, they have no money to buy the drugs. Someone had improved for the last 6 months without getting fits. Then he comes today and there is no medicine. He comes back the following day and there is no medicine. Then he fits in the compound there...That is so hurtful. All your efforts you have been putting in will be wasted. It really hurts (Mental health nurse)*.

### Multi-sectoral forums

#### South Africa

Representatives from the health sector dominated the community collaborative multi-sectoral forum, also contributing to facilitating the heightened awareness of the importance of improving mental health services evident in this sector. In addition, the forum contributed to heightened awareness amongst community representatives as reflected in the following excerpt from an interview with a community leader:

*So awareness was created at an individual level... it's (mental health) really an issue that one wasn't really bothering much about before. And even when you look at people who have got mental ill health, you wouldn't bother much... But now, this has actually conscientized us that we really have to find means and ways of helping people who have got mental health disorders...(community representative)*.

This heightened awareness at a community level translated into actual support through the provision of a community hall for the IPT depression groups.

Participation by representatives from other sectors on the multi-sectoral forum was erratic and attributed to the voluntary nature of participation, lack of seniority of representatives as well as lack of direct project activities within these sectors.

#### Uganda

In Uganda, while the multi-sectoral forum also had the greatest participation from representatives of the health sector, it did, however, manage to harness support from other sectors, especially agriculture. The Department of Agriculture provided user-carer groups with seeds and piglets to assist in their agricultural income-generating projects.

The head of District Livelihood Support Programme, also District National Agricultural Advisory Service officer attended the multi-sectoral forum meeting. He asked that the mental health focal person identifies 15 users/carers: 5 per sub-county to be supported by his programme to begin with and see how they will perform. So far, 360kgs of upland rice seeds have been availed and distributed among 10 users/carers by this programme. The rice were distributed just in time for planting as the rainy season had just began. Arrangements are underway to provide piglets as well (Minutes of multi-sectoral meeting)

### Task shifting

#### South Africa

Health managers and community members generally provided favourable responses to the concept of task shifting. Further, both psychiatric nurses and PHC nurses viewed CHWs as best placed to provide psychological interventions for CMDs given their position in the community.

*People can be trained in these issues and as for the CHWs - they are the ones that see the real problems in the community, these are often psychological- so who better to help them? We are not there in the homes of people. We can't always get to the core of the issue (Psych nurse 1)*.

*I think it is a good idea because ... they have been trained and looking at how your community mental health workers have managed to help our people... yes, yes when our patients come in... we can tell the difference. They ... say that meeting with the CMHWs has helped a lot. So I really think it can be a really good way of alleviating depression in the community (PHC nurse 2)*.

While general CHWs indicated that they did not have the time to provide specific treatment programmes such as IPT group counselling for depression, they did, however, appreciate the supportive counselling and problem management training they received. It reportedly strengthened their capacity to identify and provide counselling or referral for people with emotional problems encountered as part of their general home visitation programme.

I want to tell you that the training helped me a lot especially in the homes I visit. I used to visit homes and help them with their physical problems. Then after I received the training I learned that people have emotional problems. As a person you get depressed by your physical and emotional problems. I learned from the training how to help people with emotional problems... I know how to go beyond a physical problem when I get in the homes of people (CHW group 3)

Further, having counseling services available for CMDs is a potentially promising strategy for strengthening mental health literacy and help-seeking behavior, as reflected in the following two quotations.

*...they are in a queue say for instance talking about being depressed...You see they are sharing that experience. They seem to say ...you can go there (to the IPT groups). I've been there. You can go for yourself there and see the difference (Community leader)*.

*We found that at homes... children are being raped by their uncles and their fathers but they are scared of telling their parents. But knowing that we do counseling, they could come and tell us and then tell us not to tell their parents. I learned that most of the time children are scared of telling their parents. But in this study I found that people learn to feel free with us and reveal their secrets, even adults. Sometimes a person will say I fought with my husband and I am scared to tell people. But because you are there and you do counselling, I feel free to tell you (CMHW)*.

The importance of a supportive supervisory framework to enable task shifting to CHWs and CMHWs is, however, highlighted in the quotations below.

*It (support sessions) help us a lot. Because when we come to P (mental health counselor), we are also depressed. Even when we are at home you find that we are stressed. We come, we share, and P (mental health counselor) tries to help us so we can be free. Then we become ready to go to the community (CHW group 3)*.

I don't want to lie. When I started the sessions, I would come out very stressed. Because you would find that things that people are talking about are happening in our lives...You come to listen to people. You didn't come to tell them about your own problems... you need to be strong for these people you are helping... P (mental health counselor) would see that I am not alright...if we have been to a debriefing with P, she would ask us how was the group. We eventually got used to it. (CMHW 2)

Task shifting to PHC nurses was, however, viewed in a less positive light, with PHC nurses expressing scepticism about shifting the task of treatment of CMDs to them given that they were barely managing to cope with the current burden of physical illness.

*Those people (PHC nurses) already have too much on their plates so where will they shift their duties to or will they hire more nurses... to balance this out? (PHC nurse 2)*.

In this context, having a referral pathway for people with CMDs in the form of the mental health counselor and the trained CMHWs under her supervision, was greatly appreciated by sub-district management and PHC nurses.

*...this role has been so useful, very useful. The mental health counselor has been supporting the team here (at the sub-district hospital). The team has been working with her. They would phone each other and they would refer clients to the mental health counsellor from other clinics as well (Sub-district manager)*.

*We had the mental health counselor here ...and it made it easy when there were patients that needed mental health attention. Even when I had questions it was convenient for me because I didn't have to wait long or wait to make a call (PHC nurse 2)*.

#### Uganda

In the context of limited mental health specialist resources, task shifting was also viewed positively by district management in Uganda. It was understood to have potential to assist in increasing access to mental health services.

*I think given the resource limitation, task shifting is welcome. Because the person is again given (training) in a given component, and he can also be assigned this additional (task) to his original duties (District Health Inspector)*.

Training of the CHWs who comprise the village health teams (VHTs) was viewed as being particularly promising in that it was understood to have improved identification and referral of people with mental disorders who otherwise would not have gained access to the healthcare system.

*People have started coming up... if you had not trained the community health workers, and only trained the health workers based at the health facilities, still people would not come. They wouldn't... You have to work with the community. The moment the VHTs are functional, things become easy, because that is their role. They make sure that the patients come. They identify the patients and send them (District nursing officer)*.

In addition to increasing identification and referral, the training also emerged as a potentially useful strategy to help address stigma associated with providing care for people with mental illness amongst general health workers.

*Yes, I think it also reduced the stigma among the health workers. Because those ones who have been handling the mentally sick, they were nicknamed... like "psych" "doctor for the mentally ill" and the like. Besides, those ones who have got patients with mental illness themselves, they can now tolerate them... They no longer discriminated (against) them (Mental Health nurse 2)*.

However, training of CHWs and PHC staff was not without its problems. According to the mental health nurse, while there was an improvement in identification and referral from both CHWs and PHC staff as a result of the training, neither group assisted much with treatment. This resulted in a reported increase in the number of referrals he had to deal with. While clinical officers could prescribe medication, they were not always present in PHC clinics. Further, while PHC nurses could initiate treatment in an emergency situation, they were still required to refer patients for confirmation of the prescribed treatment. Moreover, the necessary medication was not always available.

Actually what they are doing mostly is referral. They can easily identify a person, that this one is mentally sick, and refer appropriately. That is all. They are not performing many duties... My work as a mental health worker has increased... Because at first, I had very few patients in the community, but now they are many. Because in a day, you can receive about 10 phone calls, all asking for help. (Mental Health nurse 2)

Task shifting cannot replace the need for essential psychotropic medication nor the need for a minimum number of mental health specialists. A major constraint to task shifting and the integration of mental health into PHC that emerged in Uganda was that it had to compete with other priorities in budgeting and resource allocation. This was exacerbated in the absence of ring-fencing of the mental health budget. Given more life threatening diseases, this militated against the delivery of mental health services within PHC as illustrated in the following quotation from the district nursing officer.

*I have told you how the budget is framed...stationery, outreach, what what...and when we talk of drugs, we are not talking of drugs for a particular condition. It is a mixture. That is the issue... when we are looking at like our objectives... then mental health will feature... But when it comes to the real practice, it becomes different... compared to the number of malaria cases we see in this district...hah, do you think you will see more mental cases than malaria cases? Or more than pneumonia, I don't think (so) ... (District nursing officer)*.

### Self-help groups

#### South Africa

Community level self-help groups in South Africa focused on CMDs, with CHWs using the training received to establish these groups. The majority were formed to assist HIV infected and affected women.

*We (formed this) support group of grannies who have different illnesses and who live with orphans... The grannies used to sit at home and not get up and do anything around the house. Then we talked to them. We realized that because they are living with orphans that affects them mentally. So we meet every Wednesday, they do plays, play ball and do handwork...The stress is removed (CHW group 3)*.

*There is a place we started it's a centre...We work with those people so that they share. Even if a person is scared we try and bring her... We give them a garden. We ask them to plough. At the end they eat. We cook at the centre, they eat and talk. These days we were thinking about starting handwork. If we see that someone is too depressed, we (refer). We ask them to come because they have a problem and they don't know how to solve it... Others are still scared to disclose that they have this disease (HIV). When they are all together, they share their problems, and then they realize they are not alone (CHW group 1)*.

#### Uganda

A user-carer group for people with severe mental disorders was established in Uganda. It was reported to be very beneficial for the participants, with access to medication being the initial motivation for user-carer participation. Stabilization afforded by medication was reported to then enable user engagement in other psychosocial rehabilitative activities provided by the group, which in turn assisted in promoting social inclusion and reducing stigma and discrimination.

*You know we had nothing (for treatment of) mental illness. People could not even believe it can be treated by medicine, we thought these things are related to clan, or spirits, but now they have come to know that ...there are medicines that treat such illnesses. So there is treatment now... You know we the carers, we had no hope in them but as I have seen that there is improvement - we are teaching them how to work for themselves at least to have something to do (Carer)*.

*Ok now the community... have seen the difference, we are now not ostracized as it was - because they used to not even allow our children to get close to their children, to even play with them... Stigma is no longer there in the community. They no longer point fingers (at) us...Now I have hope in my patient, which wasn't there (before). I have even taken her back to school. So now at least I am happy with the improvement (Carer)*.

In addition to assisting with the development of social skills, the group also facilitated access to resources for agricultural production e.g., rice seeds and piglets, accessed from the Department of Agriculture through the multi-sectoral forum.

*I should say that giving us the rice seeds that we received and planted recently was a good start. It was an indicator that if we continue to be united as people with a common goal, I am sure things will be much better as we go on. And I have just heard that there is something more coming up soon. So, that makes us more hopeful (User-carer group)*.

I also think if someone is going to (provide) support by equipping you with knowledge and skills, that is better than someone who just carries something physical and gives (it to) you. Because the item they give you will get finished, but the knowledge will always be there (User-carer group)

The vicious cycle of poverty and mental ill-health and the potential for these self-help groups to assist users and their carers to break this cycle through access to medication and livelihood opportunities is illustrated in the following quotation from a focus group with users and carers about how caring for a person with a mental disorder had previously affected them.

*(mental illness) has made you poor...every money you get you plan for the patient... a person does not get time to work because this illness brings a lot of distress in the family, so the patient and the caretaker both are distressed, because the moment you leave the patient for a minute you may not know where he/she has gone. You don't have time to go out to work...you spend your time watching over the patient (carer)*.

The Chairperson of the group was confident that the group would continue to be able to sustain itself independently even after the closure of the MHaPP:

*As the Chairman I think since we have started helping ourselves - being together as patients and carers ... even if this (MHaPP) no longer exists we as a group... can continue... This is how we shall handle (sustainability). If ... somebody else joins the group, we meet with the members who got ... given 2 animals - say pigs, when it delivers, he gives back 2 and these are given to another member to start so that will help to spread even if these organizations have gone*.

## Discussion

Both countries engaged in activities associated with all four aspects of the framework which served to strengthen access to mental health services, albeit the focus of the integration process differed across the two country sites.

Sensitization efforts with district/sub-district management heightened awareness of the need to dedicate more specialist mental health resources to assist with the integration of mental health into primary healthcare in both country sites. However, it was only in South Africa that this awareness translated into an actual increase in dedicated resources within the study site. This was made possible by the deployment of existing psychiatric nurses within the system. As indicated in the introduction, South Africa is better resourced than Uganda and has a relatively higher number of psychiatric nurses available in the system (see Table 1), who could be deployed to mental healthcare duties. In contrast, in Uganda, increasing the number of mental health nurses in the study site during the life of the project was not possible as this entailed a lengthy process of needing to fund and source incumbents for these specialist posts.

The community collaborative multi-sectoral forums also proved useful in both country sites for mobilizing resources for mental health. In Uganda, resources were sourced from the agricultural sector to assist members of the self-help user-carer group to engage in sustainable livelihood agricultural activities. In South Africa, community participation emerged as a useful vehicle for accessing a community hall for the IPT depression groups. The lack of formal collaborative agreements and directives from sectors other than health, however, militated against any sustainable commitments from these sectors. This suggests the need for sector-wide approaches to the development of mental health services to be initiated at higher levels within government structures if they are to be implemented at district level.

With regard to task shifting, this was viewed positively in both country sites by district/sub-district management. In South Africa, shifting psychosocial care for people with CMDs to CHWs and CMHWs was also viewed positively by PHC staff and CHWs themselves. The supportive counselling and problem management training that general CHWs received reportedly strengthened their capacity to respond to psychosocial problems and related CMDs they encountered in their regular home visits. Further, having dedicated CMHWs provide a specific psychological treatment programme for women with depression was also viewed positively as it provided a referral pathway for people identified with depressive symptoms at both the community and facility levels of PHC. PHC nurses and general CHWs reported that they could not provide these specific treatment programmes themselves, feeling overburdened already with existing duties. This corroborates previous findings [4,13]. They therefore welcomed the introduction of a referral pathway of care for these disorders. Given that CMHWs are equivalent to general CHWs in that they are community members with minimal training, they also require close supervision from mental health specialists, an essential component of the task shifting model [4]. In the demonstration project, the mental health counsellor introduced into the PHC system provided this supervisory support. This task could, however, be undertaken by another mental health specialist within the system, such as a psychiatric nurse deployed to fulfil this supportive and supervisory role.

In Uganda, where the focus was on SMDs, the training reportedly improved identification and referral of these disorders. In the absence of sufficient psychotropic medication as well as healthcare personnel who have the authority to prescribe or confirm prescriptions, task shifting reportedly had a demoralizing effect on PHC staff. A bottleneck of users requiring services from limited mental health specialists was reported. Paradoxically, instead of task shifting alleviating this problem, it was exacerbated.

Based on the two different experiences in the South African and Ugandan sites, these findings collectively suggest that for task shifting to be successful in low resource settings, it needs to occur within a stepped care approach, with adequate infrastructure and a specialist referral and supervisory support structure. Task shifting is not a panacea for the paucity of mental health specialists nor psychotropic medication in LMICs. With respect to the former, a minimum number of specialist mental health personnel are still required to provide supervision and a referral service [25]. Regarding the latter, an adequate supply of psychotropic medication at PHC level is an essential first step in the process of decentralization and re-integration of users with SMDs into society. Campaigns to raise the awareness of policy makers in LMICs and donor agencies of the need for a sufficient and constant supply of psychotropic medication need to be mounted. While this is best achieved by service users themselves, involvement of users with mental disorders in advocacy efforts is, however, difficult for a number of reasons [26], let alone in scarce resource settings where treatment options have been historically limited [27,28]. Involvement of carers and service providers in these efforts as well, is thus important.

The findings of this study suggest that when some treatment is provided (medication for SMDs in Uganda, and psychological treatment for CMDs in South Africa), help seeking behaviour is strengthened, which results in a greater demand for services. In the absence of sufficient resources, this benefit needs, however, to be weighed against the demoralizing impact it can have on service providers and users alike, as was demonstrated in the Ugandan case study site.

The form of self-help groups developed in both country sites were contextually driven by country priorities. In the context of South Africa's AIDS pandemic and in the KwaZulu-Natal province specifically, where 38.7% of childbearing women are estimated to be HIV positive [29], it was not surprising that psychosocial support groups were formed, in the main, to assist HIV infected and affected women. A recent study suggests high levels of CMDs associated with HIV (47.3%) in South Africa [30]. In Uganda, given the focus on developing services for people with SMDs, and within the context of an inconsistent supply of psychotropic medication, the initial focus of the self-help groups was on accessing medication.

Across both country sites, self-help groups generally incorporated some form of livelihood generating activities. This should assist to break the vicious cycle of poverty and mental ill health, now well established [31,32], and promote social inclusion, which in turn can assist in reducing stigma and discrimination. In Uganda, medication played an important role in this process, with the findings suggesting that engagement in livelihood generating activities by people with SMDs was initially made possible through the stabilizing effect of psychotropic medication. In the context of findings from other African countries of the high financial burden of having family members with SMDs [33], the hope amongst carers of easing this burden through participation in the self-help group, was striking.

## Conclusion

The common implementation framework helped to facilitate the integration of mental healthcare into primary healthcare across both district/sub-district country sites. This was notwithstanding the different foci and resource constraints of the two country contexts.

Key lessons for integrated primary mental healthcare in LMICs that can be derived from the two district demonstration sites include the following. First, within the context of decentralized healthcare systems and where the burden of life threatening infectious diseases is high, sensitization of district management authorities and the establishment of community collaborative multi-sectoral forums can assist to improve political will to strengthen mental health services within the healthcare sector and beyond.

Second, scaling up of mental health services can be advanced through improved mental health literacy within communities which can strengthen demand for services. This can act as a catalyst and advocacy opportunity for increasing the public health priority afforded to mental health by governments in LMICs and donor agencies. CHWs present as a potentially important resource to be harnessed for strengthening mental health literacy and help seeking behaviour for CMDs and SMDs within the task shifting model.

Third, self-help groups should be at the foreground of scaling-up efforts of mental health services in LMICs. They serve to provide treatment, rehabilitative and mental health promoting opportunities, the potential for empowering service users and carers as well as reducing dependency on overstretched healthcare systems. They also have the potential to promote social inclusion and reduce stigma, discrimination and human rights abuses of people with mental disorders.

Finally, task shifting is not a panacea for the delivery of mental health services in scarce resource contexts and limited application to identification and referral can in fact exacerbate the bottleneck of referrals to mental health specialists that task shifting attempts to alleviate. This may serve to demoralize both service providers and users alike. A stepped care approach to task shifting, as advocated by the WHO's Mental Health Gap Action Programme (mhGAP) [3], is paramount. In the absence of sufficient specialist mental health personnel, training of PHC personnel to initiate treatment should assist to alleviate this bottleneck and is more likely to ensure the availability of referral treatment services. Even this approach cannot, however, replace the need for sufficient psychotropic medication, nor the need for a minimum number of mental health specialists to provide the necessary supervisory and referral service. The latter thus still remain priorities for scaling up mental health services in LMICs, as suggested by the Lancet Global Mental Health Group [34].

## Competing interests

The authors declare that they have no competing interests.

## Authors' contributions

IP contributed to the design of the study and was responsible for the analysis of the South African data and initial draft of the manuscript. JS participated in the data collection and analysis of the Ugandan data. AB contributed to the design of the study. KB participated in the data collection and analysis of the of the South African data. All authors read and approved the final manuscript.
